# Movement behavior profiles and obesity: a latent profile analysis of 24-h time-use composition among Danish workers

**DOI:** 10.1038/s41366-019-0419-8

**Published:** 2019-07-24

**Authors:** Nidhi Gupta, David M. Hallman, Dorothea Dumuid, Akshay Vij, Charlotte Lund Rasmussen, Marie Birk Jørgensen, Andreas Holtermann

**Affiliations:** 1 National Research Centre for the Working Environment, Copenhagen, Denmark; 2 Centre for Musculoskeletal Research, Department of Occupational Health Sciences and Psychology, University of Gävle, Gävle, Sweden; 3 Alliance for Research in Exercise, Nutrition and Activity (ARENA), School of Health Sciences, University of South Australia, Adelaide, South Australia Australia; 4 Institute for Choice, University of South Australia, Adelaide, Australia; 5 Section of Social Medicine, Department of Public Health, University of Copenhagen, Copenhagen, Denmark; 6 Department of Forensic Science, University of Copenhagen, Copenhagen, Denmark; 7 Department of Sports Science and Clinical Biomechanics, University of Southern Denmark, Odense, Denmark

**Keywords:** Risk factors, Cardiovascular diseases

## Abstract

**Background/objectives:**

An element of obesity prevention is increasing total physical activity energy expenditure. However, this approach does not incorporate the balance of various movement behaviors—physical activity, sedentary behaviors and sleep—across domains of the day. We aimed to identify time-use profiles over work and leisure, termed ‘movement behavior profiles’ and to investigate their association with obesity.

**Subjects/methods:**

Eight-hundred-and-seven workers completed (a) thigh accelerometry and diaries to determine their 24-h composition of behaviors (sedentary and standing, light physical activity and moderate-to-vigorous physical activity at work and leisure, and time in bed) and (b) obesity measurements. Movement behavior profiles were determined using latent profile analyses of isometric log-ratios of the 24-h composition, and labeled according to animal movement behavior traits. Linear models were applied to determine the association between profiles and obesity.

**Results:**

Four profiles were identified, labeled as “Chimpanzees” (*n* = 226), “Lions” (*n* = 179), “Ants” (*n* = 244), and “Koalas” (*n* = 158). “Chimpanzees” work time was evenly distributed between behaviors while their leisure time was predominantly active. Compared to Chimpanzees, “Lions” were more active at work and sedentary during leisure and spent more time in bed; “Ants” were more active at work and during leisure; “Koalas” were more sedentary at work and leisure and spent similar time in bed. With “Chimpanzees” as reference, “Lions” had least favorable obesity indicators: +2.0 (95% confidence interval [CI] 0.6, 3.4) %body fat, +4.3 cm (1.4, 7.3) waist circumference and +1.0 (2.0, 0.0) Body Mass Index (BMI), followed by “Koalas” +2.0 (0.4, 3.7) %body fat, +3.1 cm (0.1, 6.0) waist circumference, and +0.8 (−0.30, 1.94) BMI. No significant differences were found between “Chimpanzees” and “Ants”.

**Conclusions:**

Movement behavior profiles across work and leisure time-use compositions are associated with obesity. Achieving adequate balance between work and leisure movement behaviors should be further investigated as a potential obesity prevention strategy.

## Introduction

The worldwide rate of obesity has tripled over the last three decades [1]. In 2016, more than 1.9 billion adults (≥18 years) were overweight, out of which around 650 million were obese [1]. Obesity is a known risk factor for diabetes mellitus, hypertension, cardiovascular diseases and mortality [2–5]. The increasing prevalence and adverse health effects of obesity necessitate more effective preventive interventions.

An existing behavioral approach to prevent obesity is to promote higher daily total energy expenditure by increasing the time spent in physical activity and reducing time spent sedentary [6]. However, this approach does not incorporate the recovery process from daily activities, shown to be of relevance for obesity [7–9]. Further, the approach of increasing total energy expenditure does not consider domains (i.e., work and leisure) of movement behaviors over the day. This could be important for obesity prevention as the pattern of movement behaviors, possibilities of variation and recovery and their health effects may depend on the domain in which they occur [10]. Therefore, an obesity prevention approach that accounts for recovery and domains of movement behaviors in a day could be useful.

An appropriate behavioral approach for obesity prevention could be to facilitate balanced distribution of time spent in movement behaviors across domains in a day. According to the principle of the *homeostasis state of the body* [11, 12], a balance can be obtained if the ‘stimulus’ on the body (e.g., physical activity) is balanced with sufficient recovery (e.g., sedentary time and sleep). However, if proper recovery is not attained, a chronic disturbed state of homeostasis and excessive cumulative allostatic load [13] can induce adverse effects such as preferential deposition of adipose tissues, promotion of energy storage as fat, and insulin resistance [14]. Similarly, too little physical activity and excessive sedentary behavior can disturb homeostasis over time, causing an energy imbalance [6]. Both of these examples of imbalanced time use over the day may contribute to obesity.

Balancing time use over a day is dependent on the inherent imposed constraints of the main domains of the day (i.e., work and leisure). For example, an office worker is likely constrained to spend most of the work time being sedentary or standing. On the contrary, a cleaner is likely constrained to do prolonged walking/standing for several hours at work [15, 16]. The office worker and the cleaner may therefore require different amounts of leisure time sedentary behavior, physical activity and sleep to achieve balanced 24-hour time use to prevent obesity.

To gain an understanding of how to best balance time spent in movement behaviors over various daily domains to prevent obesity, it may be useful to first explore time-use behavioral patterns via an exploratory person-driven approach. One such approach—latent profile analysis—detects groups with common patterns of time-use behaviors [17]. However, to date, no latent profile analysis has distinguished between work and leisure domains or accounted for the constrained nature of time use (i.e., every day can only have 24 h). It is now widely accepted that analysis of 24-hour time use should respect the compositional properties of the data [18].

Our study aimed to use compositional latent profile analysis to explore if distinct time-use profiles based on movement behaviors during work and leisure time could be identified among Danish workers, and to determine whether these profiles were associated with obesity.

## Materials/subjects and methods

This study used cross-sectional data from the Danish PHysical ACTivity cohort with Objective measurements (DPHACTO) cohort [19]. Workers from 15 companies engaged in three different sectors - cleaning, transport, and manufacturing - were recruited between December 2011 and March 2013. In total, 2107 workers were invited to participate, of which 1119 consented to participate. To be included, workers had to be able to participate during working hours. The pre-established exclusion criteria were: being in a management position, intern, pregnant and having fever on the day of testing or bandage allergy.

Data collection was conducted from spring 2012 to spring 2013. The consenting and eligible workers were invited to fill in a web-based questionnaire and to perform physical examination tests and accelerometry measurements.

All workers provided their written consent prior to participation. The present study was conducted according to the Helsinki declaration and approved by the Danish data protection agency and the local Ethics Committee (The Capital Region of Denmark, H-2-2012-011).

### Accelerometry

Workers attached a triaxial accelerometer (Actigraph GT3X+, Actigraph LLC, Florida, USA) on the right thigh for four consecutive days (4 × 24 h) including at least two working days [20]. Workers were also asked to complete a short paper-based diary noting the start and end of work periods, time in bed (going to bed and getting out of bed), non-wear time, and time of reference measurement (i.e., standing in an upright position for 15 s) during the measurement period. Workers were instructed to remove the device if it caused any kind of discomfort.

The raw data from the accelerometer were downloaded using the Actilife software (v.5, ActiGraph LLC, Pensacola, FL, USA) and later processed using a customized MATLAB program, Acti4 (The National Research Centre for the Working Environment, Copenhagen, Denmark and BAuA, Berlin, Germany). This program has shown to determine duration spent in various postures (lie, sit, and stand) and physical activity (walk, run, stair climb, and cycle) with high sensitivity (95–99%) and specificity (>99%) during standardized conditions [21]. Time spent sedentary, standing still, and in light physical activity (LIPA; including moving [standing with movements], and slow walking), MVPA (including fast walking, running, stair climbing and cycling) at work and leisure and time in bed was determined based on procedures explained elsewhere [21].

Non-wear periods were recognized according to an automatic procedure [22]. All non-wear periods and non-working days were excluded from the analyses. A day consisted of 24 h starting from midnight. A work period was defined as the self-reported work hours spent on primary occupation while the remaining hours, except time in bed, were considered as the leisure period. Time in bed, as a proxy of sleep time, was measured using information from self-reported diary that was confirmed via visual checks of accelerometry data. A day was considered valid if it contained valid work, leisure, and time in bed periods. Work and leisure periods were considered valid if they comprised at least 4 h or 75% of the worker’s average work and leisure time. Time in bed was considered valid if it was at least 4 h in duration [23]. Workers who had measurements on at least one valid day were included in further analyses.

The mean of the time spent sedentary, standing still, in LIPA, and MVPA, and a median of time-in-bed periods, across all valid days, was calculated for each worker. These summary statistics were chosen based on the distribution of the data.

### Obesity indicators

Waist circumference (WC) was measured two times horizontally midway between the top edge of the hip and lower ribs using a measurement tape (Seca, model 201) to the nearest 0.1 mm. The average of the two measurements was calculated. Weight and fat percentage (Fat%) were measured using the Tanita (model BC418 MA) bio-impedance segmental body composition analyzer [47], to the nearest 0.1 kg/0.1%. Height without shoes was measured using a stadiometer (Seca, model 213) to the nearest 0.1 cm. Body mass index (BMI) was calculated as weight (kg) divided by height (m) squared.

### Confounders

Potential confounders were chosen a priori based on previous research on the association between physical activity, sedentary behavior and sleep and obesity [7, 24, 25]. Age was determined using the workers’ unique Danish civil registration numbers. Smoking status was obtained from a single item “Do you smoke?” with four responses summarized into ‘smokers (smoking regularly, smoking occasionally)’ and ‘non-smokers (used to smoke not anymore, never smoked)’. Alcohol intake was determined using a single item “How much alcohol did you drink during the last week?” with responses in units per week. Poor dietary intake was obtained from two items; “How often do you usually eat/drink”—“Fast food, pizza, burger, shawarma, etc.?” and “Candy, ice cream, chocolate, soft drinks” with four responses (daily, 3–4/week, 1–2/week, and rarely). The responses for the two items were reversed and averaged where higher scores indicated worse dietary intake. A proxy measure of socioeconomic status was determined through a single question on predominant type of work (responses were “administration” named as “white-collar” and “production” named as “blue-collar” workers).

### Statistical analyses

All analyses were conducted in RStudio software (Version 0.99.893 – © 2009–2016) using the “Compositions” package [26] and in Mplus software [version 7.4, Muthen & Muthen [27]].

The data of time spent in all movement behaviors (sedentary, standing still, LIPA and MVPA at work and leisure, and time in bed) in a day are ‘compositional’ in nature. That is, these data exist in a constrained data space where the sum of all parts (i.e., movement behaviors in this case) always sum up to 100% (i.e., 24 h). Thus, increasing time spent in one behavior will inevitably substitute time spent in at least one other behavior within a day. This special property of the data needs to be addressed using compositional data analysis (CoDA; [28, 29]).

### Determination of movement behavior profiles and their association with obesity

First, the compositional time-use data (work [sedentary, standing still, LIPA, and MVPA] and leisure [sedentary, standing still, LIPA, MVPA], and time in bed) were expressed as a set of eight isometrical log ratios (ilrs) using the default ilr transformation in the “Compositions” R package [26]. The ilrs contain all the relative information regarding the time-use composition, and can be used as real vectors in standard statistical models instead of the compositional vectors of the raw minutes/day.

Second, the ilrs were used as inputs for compositional latent profile models. Latent profile analysis uses a finite mixture latent modeling approach to statistically derive subgroups that are homogenous with respect to their time-use behaviors within a profile and heterogeneous between profiles. To identify the best fitting latent model indicating the respective movement behavior profiles, we conducted consecutive latent models with one to five profile solutions. Then, we chose the best model based on fit statistics followed by an evaluation of the distinguished profiles according to their clinical relevance. The model fit was judged based on the following fit statistics and criteria: [1] Lo-Mendell-Rubin Test (LMRT): an inferential statistical test which compares a targeted profile solution (e.g., 5-profiles) with a 1-less profile solution (e.g., 4-profiles). We chose a 1-more profile solution if the corresponding *p*-value was less than 0.05, otherwise a 1-less profile solution was retained; [2] Akaike Information Criterion (AIC) and the Bayesian Information Criterion (BIC): these are indicators of the best balance between the simplicity of the model and the goodness of fit. Lower values indicate a more parsimonious model; [3] Entropy: ranges from 0 to 1, and describes the degree of certainty of classification of each profile solution; higher values indicate higher classification certainty; and [4] Number of workers in each group not less than 50.

Potential outliers in the multivariate compositional time-use data were identified using a principal components bi-plot [30]. Twelve outliers were identified based on a 0.999% probability ellipse. The sensitivity of the best latent profile model to these outliers was tested by determining if the decision of selecting the best latent model remained the same before and after removal of these outliers. Results indicated high agreement between the two models (Cohen’s Kappa 0.97). Thus, the outliers were kept in the dataset and subsequent analyses.

After choosing the best model, each worker was assigned to one of four profiles, based on their maximum posterior probability of being in that profile [31]. Thereafter, each profile was named to reflect similar behavioral profiles in the animal kingdom. For example, one profile was named “Lions” as the workers in this profile spent much of their work time being active (i.e., “hunting”) and most of their leisure time being sedentary or in bed.

The time-use compositions of the four movement behavior profiles were described in terms of center (compositional mean of time spent in bed and in work and leisure sedentary, LIPA and MVPA) and multivariate dispersion (variation matrix) [32].

Thereafter, we explored the linear association between membership of the movement behavior profiles (predictor) and obesity indicators (outcomes) included in separate models, adjusted for selected confounders—age, sex, smoking, alcohol, poor dietary habits and socioeconomic status. Sex, smoking, and socioeconomic status were treated as categorical variables, whilst age, alcohol intake, and poor dietary habits were treated as continuous variables.

As we did not have prior knowledge of the number of movement behaviors profiles or sufficient information about expected effect sizes of the movement behavior profiles on the obesity measures from previous studies, we could not perform an a priori sample size calculation of sufficient quality.

## Results

Of the 1119 workers who consented to participate, 807 workers provided valid accelerometry data for at least one working day, including valid periods of time in bed, work and leisure. On average, workers wore the accelerometer for 7.6 h (SD 1.2) at work and 15.8 h (SD 1.5) at leisure—including time in bed (Mean 7.0, SD 1.0)—summing to 23.4 h (SD 1.2) per day.

Table 1 shows the results of consecutive latent profile models with 1 to 5-profile solutions for 807 workers. The AIC values and entropy continued to decline from 1 to 5-profile solutions but the BIC value was the lowest at the 4-profile solution. Additionally, at the 5-profile solution, the *p*-value of LMRT became non-significant and one of the resulting profiles was very small (*n* = 8). Thus, for the further analyses, we chose the 4-profile solution based on the goodness of fit statistics and an evaluation of the biological relevance of the profiles.

**Table 1:** Fit indices of the latent 1 to 5-profile solutions (*N* = 807)

Profiles	AIC	BIC	Entropy	LMRT (*p*-value)	Min size
5	6522	6766	0.80	0.35	8
4	6541	6743	0.77	0.02	158
3	6732	6891	0.78	<0.01	210
2	7090	7208	0.82	<0.01	269
1	7949	8024	–	–	–

*AIC* akaike information criterion, *BIC* Bayesian Information Criterion, *LMRT* Lo-Mendell-Rubin Test; min size reflects the minimum size of the profile in each solution

**Fig. 1:**
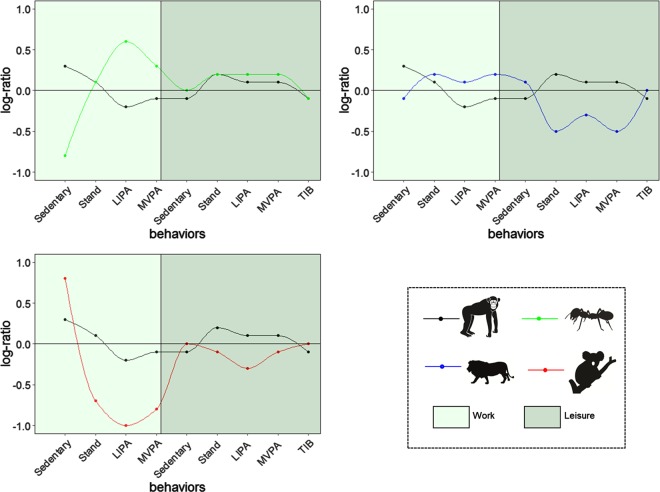
Compositional Profile Plots indicating the relative difference between time-use compositional means of each profile compared to the whole population. The profiles for Lions, Koalas and Ants are compared with Chimpanzees (black). Values on the Y axis represent the log of the ratio between a particular profile mean and the population mean. A score of 0 for any behavior means that, on average, equal time is spent in that behavior by the whole population as by the profile of interest. Positive and negative values represent higher and lower compositional means than the whole population. On the basis of the log-ratio value, the actual ratio between the composition mean of a particular profile and whole population can be calculated. For example the log-ratio value for sedentary time at work for Koalas is 0.81. This means that on average, Koalas are 2.3 times more (exp(0.81)) sedentary at work compared to the whole population. *LIPA* light physical activity, *MVPA* moderate to vigorous physical activity, *TIB* time in bed

Figure 1 and Table 2 show the compositional mean of the daily time-use composition for the four movement behavior profiles. We named the four movement behavior profiles as “Chimpanzees”, “Lions”, “Koalas” and “Ants”. We considered the “Chimpanzees” profile to have the most balanced movement behavior profile across the different domains of the day and used this profile as a reference for comparisons.

**Table 2:** Demographical, lifestyle, obesity and work-related descriptive statistics for the total population and the four movement behavior profiles

Variables	Total (*n* = 807)				Chimpanzees (*n* = 226)				Lions (*n* = 179)				Koalas (*n* = 158)				Ants (*n* = 244)			
	*n*	x̅	SD	%	*n*	x̅	SD	%	*n*	x̅	SD	%	*n*	x̅	SD	%	*n*	x̅	SD	%
Demographic variables																				
Age (years)	807	45.1	9.7		226	44.0	9.0		179	43.4	11.3		158	45.7	8.7		244	47.0	9.5	
Male	439			54.4	144			63.7	130			72.6	87			55.1	78			32.0
Lifestyle-related variables																				
Alcohol intake (units/week)	761	4.5	6.0		216	4.3	5.1		171	5.1	6.7		152	4.8	6.0		222	3.9	6.3	
Poor diet (0–3)*	792	1.8	1.0		222	1.9	1.1		177	1.7	1.0		157	2.1	1.0		236	1.8	1.1	
Smokers	216			27.4	50			22.7	62			35.2	33			21.2	71			30.2
Obesity Health-related variables																				
BMI (kg/m^2^)	790	27.2	4.8		221	26.9	4.6		172	27.8	5.1		157	27.0	4.4		240	27.3	4.8	
WC (cm)	570	93.3	12.6		171	91.7	12.6		110	96.6	12.2		134	92.9	13.0		155	93.1	12.3	
Fat (%)	790	29.1	9.3		222	27.0	9.2		175	27.8	9.5		157	29.0	7.9		236	32.2	9.5	
Work-related variables																				
Blue-collar workers	671			83.1	196			86.7	173			96.6	61			38.6	241			98.8
Compositional means of sedentary behaviors, standing still, LIPA, and MVPA at work and leisure and time in bed (total = 1440 min)																				
Sedentary behavior at work		149				197				137				333				64		
Standing still at work		137				145				167				68				156		
LIPA at work		70				60				79				26				123		
MVPA at work		60				57				76				27				79		
Sedentary behavior at leisure		353				306				380				346				342		
Standing still at leisure		103				121				62				95				122		
LIPA at leisure		57				62				43				43				70		
MVPA at leisure		45				52				28				39				52		
Time in bed		466				440				468				462				432		

*LIPA* light physical activity, *MVPA* moderate-to-vigorous physical activity, *WC* waist circumference, *BMI* body mass index; *0 = never, 3 = everyday; x̅ = mean

The “Chimpanzees” profile (*n* = 226, 28% of the sample) was characterized by (i) a rather evenly distributed composition of work behaviors (sedentary = 197 min; standing = 145 min; physical activity = 117 min), (ii) an active leisure time (physical activity = 114 min; standing = 121 min), and (iii) 440 min in bed.

Compared to the “Chimpanzees” profile, “Lions” (*n* = 179, 22% of the sample) were characterized by (i) more physical activity and standing at work, (ii) more sedentary behavior and less physical activity at leisure, and (iii) slightly more time in bed;

“Ants” (*n* = 244, 30% of the sample) were characterized by (i) more physical activity and standing at work, (ii) almost similar physical activity and standing but higher sedentary behavior at leisure, and (iii) similar time in bed.

“Koalas” (*n* = 158, 20% of the sample) were characterized by (i) more sedentary behavior and less physical activity and standing at work, (ii) higher sedentary behavior and less physical activity and standing at leisure (iii) slightly higher time in bed.

Table 2 shows the distribution of demographical, lifestyle, health and work-related indicators across the four movement behavior profiles. On average, workers in the “Chimpanzees” profile were 44 years old, 64% of them were men and 87% of them were blue-collar workers. They consumed 4 units of alcohol per week, 40% of them reported to consume a poor diet ‘everyday’ and 23% reported themselves to be smokers.

Compared to “Chimpanzees”; “Lions” were younger, included more men, blue-collar workers and smokers, had a lower prevalence of poor dietary intake and alcohol intake; “Ants” were older, included more women, fewer smokers; “Koalas” were older, included more women and white collar workers, fewer smokers, had a higher prevalence of poor dietary intake and alcohol intake.

Table 3 shows the linear associations between the four movement behavior profiles and obesity indicators, adjusted for potential confounders. Compared to “Chimpanzees”; “Lions” had significantly higher BMI (kg/m^2^; +1.0; 95% CI 0.1, 2.0), WC (cm; +4.3; 1.4, 7.3), and fat% (%; +2.0; 0.6, 3.4); “Koalas” also had higher obesity indicators (WC, +3.1; 0.1, 6.2, fat%, +2.0; 0.4, 3.7), however the results were non-significant for BMI. In our sample, “Ants” had slightly higher obesity indicators than “Chimpanzees”, but the differences were not statistically significant.

**Table. 3:** Multiple linear regression analysis of the association between the four movement behavior profiles and obesity indicators among 807 workers

Variables	Profile	β	95% CI	*p*-value
BMI (kg/m^2^)	Chimpanzees	Ref		
	Lions	**1.0**	**0.1, 2.0**	**0.04**
	Koalas	0.8	−0.3, 1.9	0.15
	Ants	0.4	−0.5, 1.3	0.39
	Chimpanzees	Ref		
WC (cm)	Lions	**4.3**	**1.4, 7.2**	**<0.001**
	Koalas	**3.1**	**0.1, 6.2**	**0.05**
	Ants	2.6	−0.2, 5.3	0.07
	Chimpanzees	Ref		
Fat (%)	Lions	**2.0**	**0.6, 3.4**	**0.01**
	Koalas	**2.0**	**0.4, 3.7**	**0.02**
	Ants	1.0	−0.4, 2.4	0.16

Each model is adjusted for age, sex, smoking status, alcohol, poor dietary intake, and socioeconomic status; *BMI* body mass index, *WC* waist circumference, *CI* confidence interval; “Chimpanzees” was set as a reference; results in bold are significant at *p* < 0.05; β is the beta estimate that indicated estimated difference in obesity indicators between each animal profile and “Chimpanzees”

## Discussion

This is the first study to explore latent movement behavior profiles based on 24-h time-use compositions among a working population. Most similar previous studies (a) have been performed on children and adolescents [33–36] or (b) did not use 24-h data [37–39] or (c) did not use device-based measures of movement behaviors [37, 38]. We found that workers in this study could be divided into four distinct movement behavior profiles, that we named “Chimpanzees”, “Lions”, “Koalas”, and “Ants”.

Compared to the remaining movement behavior profiles, “Chimpanzees” were characterized by (a) relatively moderate occupational physical activity, (b) high leisure-time physical activity, and (c) slightly less time in bed. Although “Chimpanzees” had total time in physical activity similar to “Lions” and less than “Ants”, we theoretically considered this profile to have the most balanced time-use composition. For instance, moderate (≈50% of the time) physical activity at work would have given “Chimpanzees” recovery opportunities and lowered the likelihood of work-related fatigue, thereby requiring less recovery time to obtain homeostasis. Additionally, they spent more time in physical activity during leisure, which has shown numerous health benefits, including weight loss [40]. In agreement, we found that “Chimpanzees” had the most favorable obesity indicators compared to the three other profiles. On average, “Chimpanzees” had 27 kg/m^2^ (SD = 5) BMI, 92 cm (SD = 13) WC, and 27% (SD = 9) fat%. These results were robust even after adjusting for several potential confounders including age, gender, smoking, alcohol intake, poor dietary habits, and socioeconomic status. However, other factors such as psychosocial factors and total energy expenditure may have played a role that needs to be explored in future studies.

As expected, “Koalas” had clinically relevant higher obesity levels [41] compared with “Chimpanzees”. These results may be explained by their time-use profile. Compared to “Chimpanzees”, “Koalas” were more sedentary and less physically active both at work and in leisure, and spent slightly more time in bed. This profile could be considered to be the least favorable for obesity prevention because of (a) higher sedentary time and less physical activity resulting in lower energy expenditure, and (b) the balance-driven approach (i.e., because of an imbalance in time spent in physical activity, sedentary behavior and sleep time [recovery] throughout a day).

Although not statistically significant in our sample, “Ants” had 2.6 cm higher WC, 1.0% higher fat% and 0.4 kg/m^2^ higher BMI than “Chimpanzees”. We advocate similar future studies to confirm these results. Theoretically, if this difference was statistically significant, these results contradict the traditional obesity prevention approach advocating increasing total physical activity levels. Since “Ants” had overall higher total physical activity levels and consequently higher energy expenditure, this profile would be considered to be the most favorable profile for obesity prevention. However, this was not the case in our study. Our results rather would suggest that a predominance of physical activity and limited time to recover (by being sedentary and by spending more time asleep) might hamper homeostasis, thus not being optimal for preventing obesity. “Ants” were mostly females and thus genetically predisposed to higher fat%. However, adjusting for gender did not change our results. This result thus supports the premise that the profile of movement behaviors throughout the day is of relevance for obesity. However, as the difference between “Chimpanzees” and “Ants” was not statistically significant, and the results are based on cross-sectional analyses, more similar research is needed to confirm these findings in larger prospective cohorts.

Compared to “Chimpanzees”, “Lions” had clinically relevant higher obesity levels —1.0 kg/m^2^ higher BMI, 4.3 cm higher WC, and 2.0% higher fat%. Overall, “Lions” total duration spent in LIPA and MVPA during a whole day—226 min— was in fact similar to “Chimpanzees”—231 min. Therefore, according to the traditional obesity prevention approach, both profiles would be expected to have similar obesity levels. However, this was not the case. These results may rather favor the proposed balance-driven approach to prevent obesity. Compared to “Chimpanzees”, “Lions” performed more physical activity at work (~70% of the worktime), perhaps giving them less opportunity to recover at work. This may explain why they spent more time being sedentary during leisure (≈74% of waking time at leisure), and slightly more time in bed. “Lions” accrued most of their physical activity at work and recent evidence shows that work-time physical activity does not produce the same health benefits as leisure-time physical activity [10, 42]. Leisure time physical activity is characterized by voluntary dynamic movements at conditioning intensity levels sufficient to improve cardiorespiratory fitness and metabolism, promoting obesity reduction [10]. In contrary, occupational physical activity is characterized by static load, heavy lifting, and monotonous postures, generally not conducted at sufficient conditioning intensity and performed for longer periods with fewer recovery breaks [10], being less efficient for preventing obesity. More research of detailed profiles based on intensity, bout durations and frequency of occupational and leisure-time movement behavior in relation to obesity are needed to confirm these results.

### The implication of the findings

The results suggest that obtaining a balance between time spent in physical activity over the main domains of the day whilst allowing for recovery (sedentary and bedtime), ought to be considered as a potential obesity prevention approach.

### Strengths and limitations

This study has several important strengths. First, time-use behaviors were derived from posture-identification instead of using arbitrary thresholds based on counts per minute that are criticized for not accurately differentiating between sedentary and standing postures [43]. Time spent in various movement behaviors was determined using Acti4 software [21]. Acti4 has shown to identify postures and physical activities with high sensitivity (80%) and specificity (>90%) during semi-standardized and free-living conditions [21, 44]. Second, accelerometer wear-time was high in this study. The average wear time was 23.4 h (SD 1.2), unlike previous studies where participants had to remove accelerometers during showering or swimming leading to reduced wear time [45, 46]. Third, the use of CoDA enabled the compositional nature of 24-hour time-use data to be respected in the analysis. Fourth, we obtained domain-specific behavior information, as movement behaviors may contribute differently to obesity depending on the domain they occur in [20]. Finally, the study used latent profiling analysis which resulted in meaningfully interpretable clusters based on multivariate data.

One limitation of the study is the cross-sectional design which cannot reveal the causality between profiles and obesity and thus the results of our study need to be verified using a prospective study design. Another limitation is the inability to adjust the results for total energy expenditure. In addition, we did not have an external dataset to allow us to determine the external validity of these profiles. Therefore, similar future studies should be conducted on other datasets to determine if these four profiles exist in other working populations, and if they are similarly associated with obesity. As this study included workers from cleaning, manufacturing and transport sector only, our findings may be generalized to these sectors only.

## Conclusion

In conclusion, participants in this study could be grouped into four movement behavior profiles that we named “Chimpanzees”, “Lions”, “Koalas”, and “Ants”. “Chimpanzees” had the most balanced time-use profile. Compared to “Chimpanzees”, “Lions” were highly physically active at work, more sedentary at leisure and had slightly more time in bed; “Koalas” were more sedentary at both domains and had slightly higher time in bed; “Ants” were physically active in both domains while spending similar time in bed. The movement behavior profiles were associated with obesity. Compared to “Chimpanzees”, “Lions” had the least favorable obesity indicators followed by “Koalas” and “Ants”. An approach of obtaining a balance between physical activity stimulus and recovery at work and leisure may be promising for obesity prevention instead of only focusing on increasing physical activity or reducing sedentary behavior.

## Data Availability

The R codes for our analyses are available upon request.
